# Zika Virus Replication in a Mast Cell Model is Augmented by Dengue Virus Antibody-Dependent Enhancement and Features a Selective Immune Mediator Secretory Profile

**DOI:** 10.1128/spectrum.01772-22

**Published:** 2022-07-05

**Authors:** Jeremia M. Coish, Robert W. E. Crozier, John S. Schieffelin, Jens R. Coorssen, Fiona F. Hunter, Adam J. MacNeil

**Affiliations:** a Department of Health Sciences, Brock University, St. Catharines, Ontario, Canada; b Section of Pediatric Infectious Disease, Department of Pediatrics, Tulane University School of Medicine, New Orleans, Louisiana, USA; c Department of Biological Sciences, Brock University, St. Catharines, Ontario, Canada; Regional Centre for Biotechnology

**Keywords:** CD32, Fc gamma RII, Zika virus, antibody-dependent enhancement, basophil, chemokines, cytokines, dengue virus, mast cell

## Abstract

Zika virus and dengue virus are evolutionarily related and structurally similar mosquito-borne *Flaviviruses*. These congruencies can lead to cross-reactive antibody binding, whereby antibodies generated from previous dengue virus immunity can augment Zika virus replication *in vitro*. This phenomenon, termed antibody-dependent enhancement, may participate in the clinical manifestations detected in areas with *Flavivirus* cocirculations where Zika virus is endemic; however, a causal relationship has yet to be determined. The KU812 mast cell/basophil line was integral in identifying the first *Flavivirus* infection in mast cells and serves as an effective *in vitro* model to study dengue virus antibody-dependent enhancement. Mast cells, sentinel white blood cells intrinsic in coordinating early immune defenses, are characteristically situated in the intradermal space and are therefore among the first immune cells interfaced with blood-feeding mosquitoes. Here, we tested whether KU812 cells were permissive to Zika virus, how previous dengue virus immunity might augment Zika virus infection, and whether either condition induces an immunological response. We report an antibody-dependent enhancement effect of Zika virus infection in KU812 cells across multiple time points (48, 72, and 96 hours postinfection [hpi]) and a range of multiplicities of infection (4.0 × 10^−3^ to 4) using various concentrations of cross-reactive dengue virus monoclonal antibodies (D11C and 1.6D). This antigen-specific antibody-mediated infection was selectively coupled to chemokine ligand 5 (CCL5), interleukin 1β (IL-1β), and C-X-C motif chemokine ligand 10 (CXCL10) secretion and a reduction in granzyme B (GrB) release. Therefore, mast cells and/or basophils may significantly augment Zika virus infection in the context of preexisting dengue virus immunity.

**IMPORTANCE** Antibodies generated against one dengue serotype can enhance infection of another by a phenomenon called antibody-dependent enhancement (ADE). Additionally, antigenic similarities between Zika and dengue viruses can promote Zika virus infection by way of ADE *in vitro* using these very same anti-dengue antibodies. We used the KU812 cell line to demonstrate for the first time that anti-dengue antibodies enhanced infectious Zika virus replication in a mast cell model and specifically increased CCL5, CXCL10, and IL-1β, while also impairing granzyme B secretion. Furthermore, enhanced Zika virus infection and selective mediator release were mechanistically dependent on fragment crystallizable gamma receptor II (FcγRII). These findings establish a new model for Zika virus research and a new subcategory of immune cells previously unexplored in the context of Zika virus enhancement while being some of the very first immune cells likely to meet a blood-feeding infected mosquito.

## INTRODUCTION

Antibody-dependent enhancement (ADE) is a phenomenon whereby preexisting humoral immunity to one viral infection may augment infection of a subsequent antigenically similar virus. Enhanced viral replication in an antibody-dependent mechanism was first observed *in vitro* with Murray Valley encephalitis virus (1). It was not until Halstead et al. (2) reported a similar *in vitro* observation with dengue virus (DENV) that ADE would begin to gain significant attention. DENV-enhanced infection is a rare idiosyncrasy in which preexisting immunity to a particular DENV serotype can enhance disease severity to a heterotypic secondary DENV infection. However, ADE is a controversial phenomenon in part due to challenges in studying its impact, including a historical lack of long-term epidemiological studies and, at times, improper attribution of *in vitro* studies to humans. It was not until large epidemiological studies in children that evidence of ADE of DENV was uncovered (3, 4).

Zika virus (ZIKV) infection is associated with fever, muscle aches, conjunctivitis, Guillain-Barré, spontaneous abortion, microcephaly, and intrauterine growth restriction (5). Considering the “double-tap” potential of DENV infection, the impact of previous DENV immunity on ZIKV infection severity gained significant attention during the 2015 Latin America ZIKV epidemic with reports of potential anti-DENV antibodies cross-reacting with ZIKV (6–9). ZIKV and DENV are mosquito-borne *Flaviviruses* that have significant evolutionarily conserved parallels (10). Amino acid similarities between DENV and ZIKV E structural proteins (7, 11) bridge the immune response where DENV antibodies have the potential to cross-react with ZIKV at nonneutralizing levels and augment ZIKV replication by ADE *in vitro* (8, 12–15) and *in vivo* (12, 16).

It was later suggested that the severity of the Latin American ZIKV epidemic was in part attributable to sero-cross-reactivity complexes that augmented ZIKV infection in areas of *Flavivirus* endemicity (7, 8, 15, 17). However, studies have also shown cross-protection of ZIKV infection by DENV humoral responses (7, 9, 18, 19) along with minimal impact on enhancement or neutralization at convalescence (20). Rigorous long-term prospective epidemiological studies are needed to determine the extent of flavivirus cross-reactivity to which rationale should be supported from *in vitro* and *in vivo* discoveries.

Mast cells are sentinel leukocytes that generate expulsive physiological reactions against helminths, along with inappropriate immune responses to innocuous substances clinically characterized as allergies (21). Additionally, mast cells are integral in coordinating early immune defenses as they continuously surveil the connective tissue and mucosal barriers for pathogenic microorganisms (22). Characteristically situated in the periphery, cutaneous mast cells are then among the first immune cell types interfaced with an infectious mosquito at the intradermal space. Mast cell-mosquito interactions are most commonly experienced as a classical wheal and flare reaction (i.e., “mosquito bite”) as a result of mast cell-immune mediated responses to mosquito salivary proteins that aid in the blood feeding process (23). Phenotypically similar to the mast cell is the KU812 mast cell/basophil precursor (24, 25), a cell model consistently used to explore mast cell-*Flavivirus* interactions (26–34). Additionally, this cell type has been shown to be permissive to DENV infection in an ADE model, greatly contributing to our understanding of mast cell-*Flavivirus* interactions (26, 28, 32). Mast cells were first implicated in DENV infection given that many of their classically attributed vasoactive secreted mediators contribute to DENV-associated vascular leakage pathologies. Mast cells have been shown to be the most important source of newly emerged DENV virions, along with the release of host immune factors (35). Coupled with the fact that the human skin represents the initial location of virus replication before reaching deeper organs (35), this prompted us to explore whether mast cells could also be contributors to ZIKV replication. We tested whether the KU812 cell line is permissive to ZIKV infection, how DENV immunity might augment ZIKV infection *in vitro*, and how the release of immunological mediators compares in both conditions. Recently, the first evidence of mast cell infection by ZIKV emerged from both our group (36) and Rabelo et al. (37), who report that placental mast cells and the HMC-1 mast cell line are permissive to ZIKV. We employed plaque assays for quantification of productive viral replication (i.e., infectious replication) in the previously unexplored KU812 cell model to further investigate ZIKV infection of mast cells. Additionally, we established that cross-reactive DENV antibodies enhance ZIKV infection in KU812 cells, which is selectively coupled to enhanced chemokine ligand 5 (CCL5), interleukin 1β (IL-1β), and C-X-C motif chemokine ligand 10 (CXCL10) secretion and inhibited granzyme B (GrB) secretion. These results indicate that cross-reactive antibodies generated from a DENV exposure may enhance ZIKV infection via mast cell-host interactions, offering new insights into the potential mechanisms of ZIKV infection, replication kinetics, and immune responses.

## RESULTS

### KU812 infection by Zika virus is augmented by dengue virus-specific antibody-dependent enhancement.

To determine the extent of productive ZIKV replication, KU812 cells were incubated with ZIKV across a range of multiplicities of infection (4.0 × 10^−3^ to 4) and DENV human monoclonal antibody (hMAb) concentrations (6, 10, 12 μg/mL) and examined at various times postinfection (4, 48, 72, and 96 hours postinfection [hpi]). Cell-free supernatants were collected, and viral titers were quantified by plaque assay.

**FIG 1:**
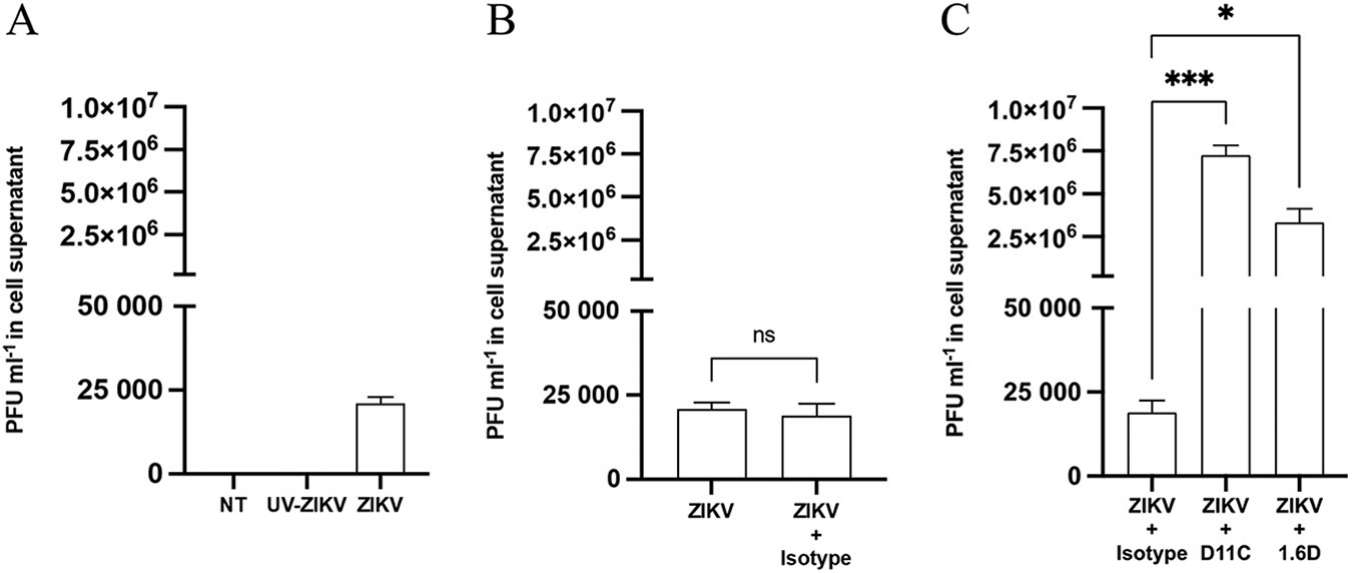
Cross-reactive dengue virus antibodies augment infectious Zika virus replication in KU812 cells at 72 h. (A) KU812 cells were infected with ZIKV-PRVABC59 (MOI, 1) or inactivated UV-ZIKV for 1 h. Cell-free supernatants were collected 72 hpi, and production of active, infectious virus was quantified by plaque assay. Data are expressed as PFU/mL ± standard error of the mean (SEM) for *n* = 3 independent experiments, each replicated in duplicate. (B) KU812 cells were exposed to ZIKV-PRVABC59 (MOI, 1) for 1 h in the presence or absence of a nonspecific isotype control antibody (10 μg/mL). Cell supernatants were collected 72 hpi, and virus production of active, infectious virus was quantified by plaque assay. A paired Student’s *t* test was performed to determine statistical significance. (C) KU812 cells were infected with ZIKV-PRVABC59 (MOI, 1) for 1 h in the presence of a nonspecific isotype control antibody, DENV-specific D11C, or 1.6D (10 μg/mL). Cell-free supernatants were collected 72 hpi, and production of active, infectious virus was quantified by plaque assay. A one-way ANOVA and Dunnett’s multiple comparison were used to determine whether the presence of DENV antibodies enhanced ZIKV replication compared to that of the nonspecific isotype control. Data are expressed as PFU/mL ± SEM for *n* = 3 independent experiments, each replicated in duplicate. *, *P* < 0.05; ***, *P* < 0.001; ns, nonsignificant.

Initially, cells were infected at a multiplicity of infection (MOI) of 1 and collected 72 hpi (Fig. 1). A viral titer of 10^3^ to 10^4^ PFU/mL was quantified from the supernatants of cells incubated with ZIKV only (Fig. 1A), whereas viral titers were not detected under negative-control conditions (no treatment [NT] and ultraviolet-Zika virus [UV-ZIKV]). Additionally, there were no differences in viral titers between cells infected with ZIKV alone and those infected in the presence of a nonspecific human isotype control IgG antibody (*P* > 0.05) (Fig. 1B). However, ZIKV incubated with DENV hMAbs D11C and 1.6D (10 μg/mL) prior to cell adsorption resulted in a >200-fold increase in infectious units per milliliter of cell supernatant compared to that of the use of a nonspecific human isotype control IgG antibody (*P* values of <0.001 and 0.05) (Fig. 1C).

**FIG 2:**
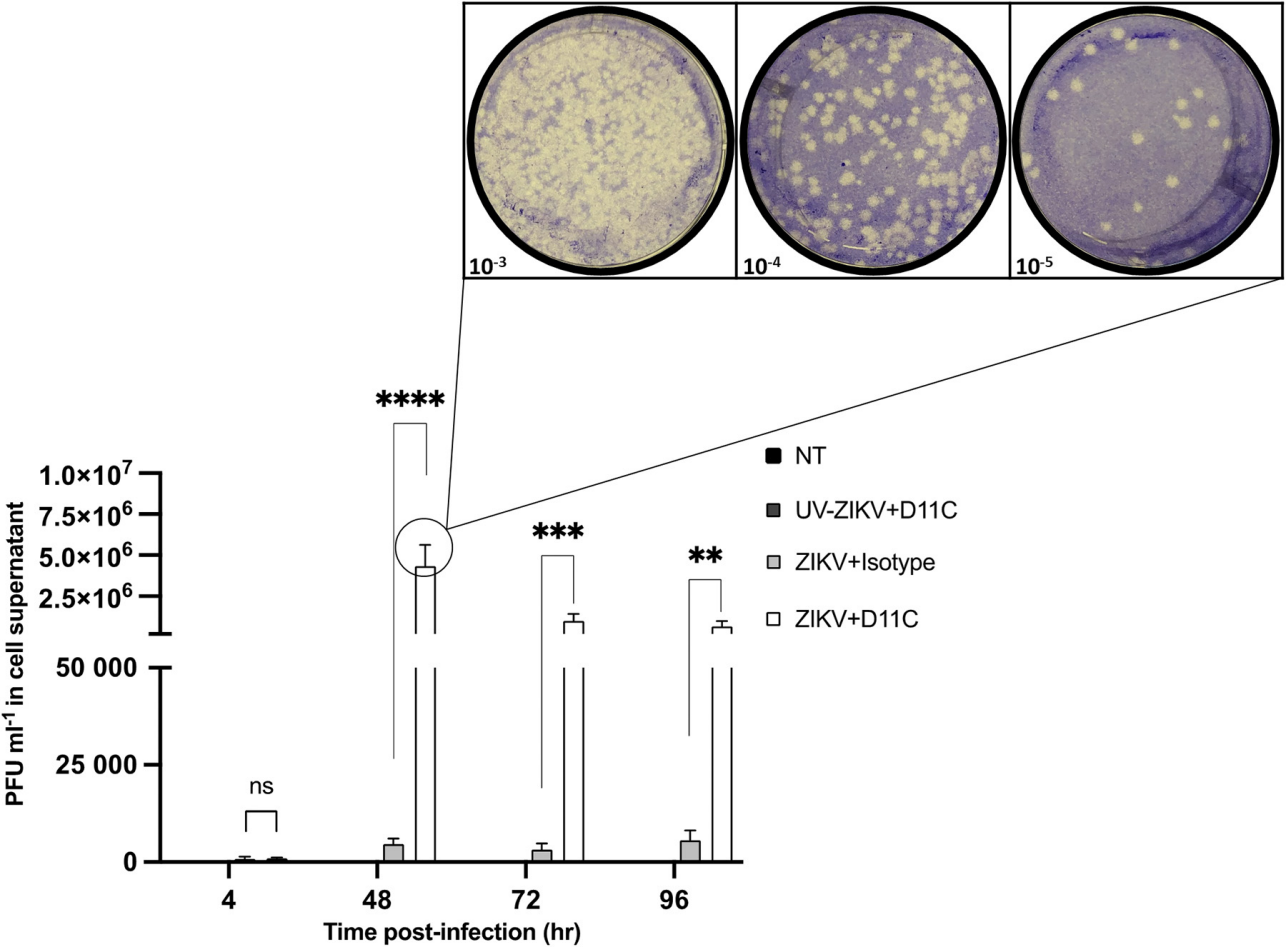
Cross-reactive dengue virus D11C antibodies augment Zika virus replication in KU812 cells across time. KU812 cells were incubated with ZIKV-PRVABC59 (MOI, 1) for 1 h with either 6 μg/mL D11C or a nonspecific isotype control antibody, washed, and resuspended with fresh growth media. Cell-free supernatants were collected 4, 48, 72, and 96 hpi. Infectious virus was quantified by plaque assay. A two-way ANOVA and a Tukey’s multiple-comparison test were used to determine whether the presence of D11C enhanced ZIKV replication compared to that of the nonspecific isotype control. Data are expressed as PFU/mL ± SEM for *n* = 3 independent experiments, each replicated in duplicate. **, *P* < 0.01; ***, *P* < 0.001; ****, *P* < 0.0001; ns, nonsignificant.

Subsequently, enhanced ZIKV replication was achieved with a decrease in D11C concentration (6 μg/mL) across multiple time points (Fig. 2). Cell supernatants were collected at 4, 48, 72, and 96 hpi. At 48 hpi, productive viral replication of cells that were infected in the presence of D11C was significantly greater than that of the isotype control (*P* < 0.0001). At 72 hpi, productive replication was still significantly higher than that of the isotype control (*P* < 0.001). By 96 hpi, viral titers were lower than those at 48 and 72 hpi, yet still significantly greater than those of the control (*P* < 0.01). At 4 hpi, there was no difference in ZIKV titers between KU812 cells infected through D11C antibody complexes and cells infected with the control antibody (*P* > 0.05).

**FIG 3:**
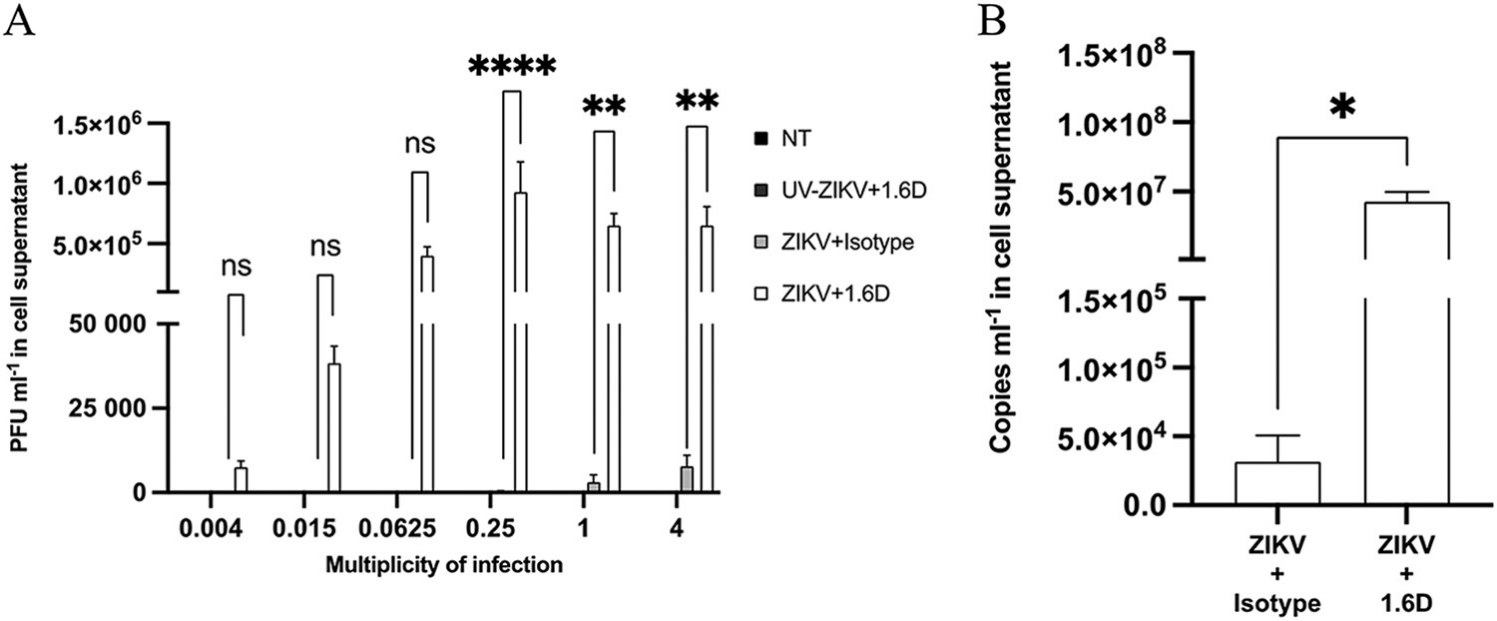
Cross-reactive dengue virus 1.6D antibodies augment Zika virus replication in KU812 cells across different multiplicities of infection. (A) KU812 cells were incubated with ZIKV-PRVABC59 (MOI, 4.0 × 10^−3^ to 4) for 1 h with either 6 μg/mL 1.6D or a nonspecific isotype, washed, and resuspended with fresh growth media. Cell-free supernatants were collected at 72 hpi, and infectious virus was quantified by plaque assay. A two-way ANOVA and a Tukey’s multiple-comparison test were used to determine whether the presence of 1.6D enhanced ZIKV replication compared to that of the nonspecific isotype control. Data are expressed as PFU/mL ± SEM for *n* = 3 independent experiments, each replicated in duplicate. **, *P* < 0.01; ****, *P* < 0.0001; ns, nonsignificant. (B) Viral RNA was extracted and then quantified by qPCR to determine the number of ZIKV copies. A two-tailed paired Student’s *t* test was used to determine whether augmented ZIKV infection resulted in an increase in RNA copies. Data are expressed as PFU/mL ± SEM for *n* = 3 independent experiments, each replicated in duplicate. *, *P* < 0.05.

At 72 hpi, ZIKV replication was also enhanced with DENV antibody 1.6D (6 μg/mL) across a range of multiplicities of infection tested (4.0 × 10^−3^ to 4) (Fig. 3A). Compared to that of cells in isotype-controlled conditions, ZIKV replication was significantly augmented (10^5^ to 10^6^ PFU/mL) in KU812 cells that were infected with an MOI equal to 0.25 (*P* < 0.0001), 1 (*P* < 0.01), and 4 (*P* < 0.01). When MOI was 0.0625 or below, ZIKV infection was not statistically significant relative to the isotype control. Additionally, quantitative PCR (qPCR) analysis was performed on viral supernatants from cells infected at an MOI of 1 to confirm the presence of ZIKV genome (Fig. 3B). There was a significant increase in ZIKV copies per milliliter in the viral supernatant of KU812 cells infected under enhanced conditions (10^7^ copies/mL) relative to that of KU812 cells infected in the absence of 1.6D (10^4^ copies/mL) (*P* < 0.05).

### Antibody-dependent enhancement of Zika virus by cross-reactive dengue virus antibodies is dependent on FcγRII receptors.

**FIG 4:**
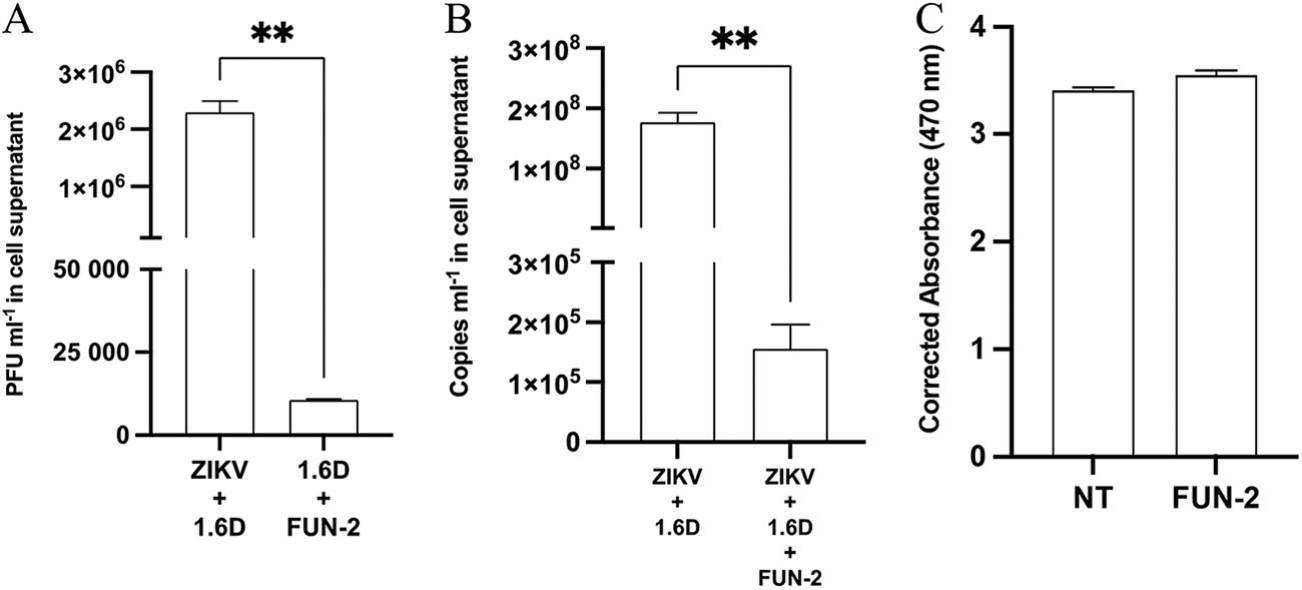
Antibody-dependent enhancement of ZIKV by DENV antibodies in KU812 cells is dependent on FcγRII. (A) KU812 cells were incubated with an FcγRII block FUN-2 (50 μg/mL) for 1 h at 37°C and 5% CO_2_. KU812 cells were then incubated with ZIKV-PRVABC59 (MOI, 1) for 1 h with 1.6D (12 μg/mL), washed, and resuspended with fresh growth medium. Cell supernatants were collected at 72 hpi, and infectious virus was quantified by plaque assay. A two-tailed paired Student’s *t* test was used to determine whether FUN-2 inhibited antibody-dependent enhancement of viral replication. Data are expressed as PFU/mL ± SEM for *n* = 3 independent experiments, each replicated in duplicate. **, *P* < 0.01. (B) Viral RNA was extracted and then quantified by qPCR to determine the number of ZIKV copies. A two-tailed paired Student’s *t* test was used to determine whether augmented ZIKV infection resulted in an increase in RNA copies. Data are expressed as PFU/mL ± SEM for *n* = 3 independent experiments, each replicated in duplicate. **, *P* < 0.01. (C) KU812 cells 2 × 10^5^/well were incubated at 37°C with FUN-2 (50 μg/mL) in 100 μL final volume for 1 h. After 1 h, 10 μL/well of WST-1 cellular proliferation reagent was added and again incubated for 4 h, and then analyzed spectrophotometrically. A two-tailed paired Student’s *t* test was used to determine whether there was a difference between the groups. Data are expressed as corrected absorbance ± SEM for *n* = 3 independent experiments, each replicated in duplicate.

Antibody-dependent enhancement of ZIKV in KU812 cells was inhibited using an anti-CD32 (fragment crystallizable gamma receptor II; FcγRII) antibody (FUN-2) (Fig. 4A). To demonstrate the inhibitory capacity of CD32 blocking, we infected KU812 cells with ZIKV coupled to 1.6D antibodies (12 μg/mL) as described in the literature (15) to produce robust infection and collected supernatant 72 hpi. When CD32 receptors were blocked, viral titers were significantly lower than those of KU812 cells that were not blocked (*P* < 0.01). Additionally, ZIKV genomic copies from blocked cells (10^5^ copies/mL) were significantly lower than ZIKV copies quantified in the ZIKV-enhanced group (10^8^ copies/mL) (*P* < 0.01) (Fig. 4B). This drop in viral titer was not attributed to cell death, as no difference in cell viability was detected with cells incubated with FUN-2 compared to that of cells in the absence of FUN-2 (*P* > 0.05) (Fig. 4C).

### IL-1β, CXCL10, and CCL5 secretion is augmented through an antibody-dependent mechanism, whereas granzyme B secretion is inhibited.

**FIG 5:**
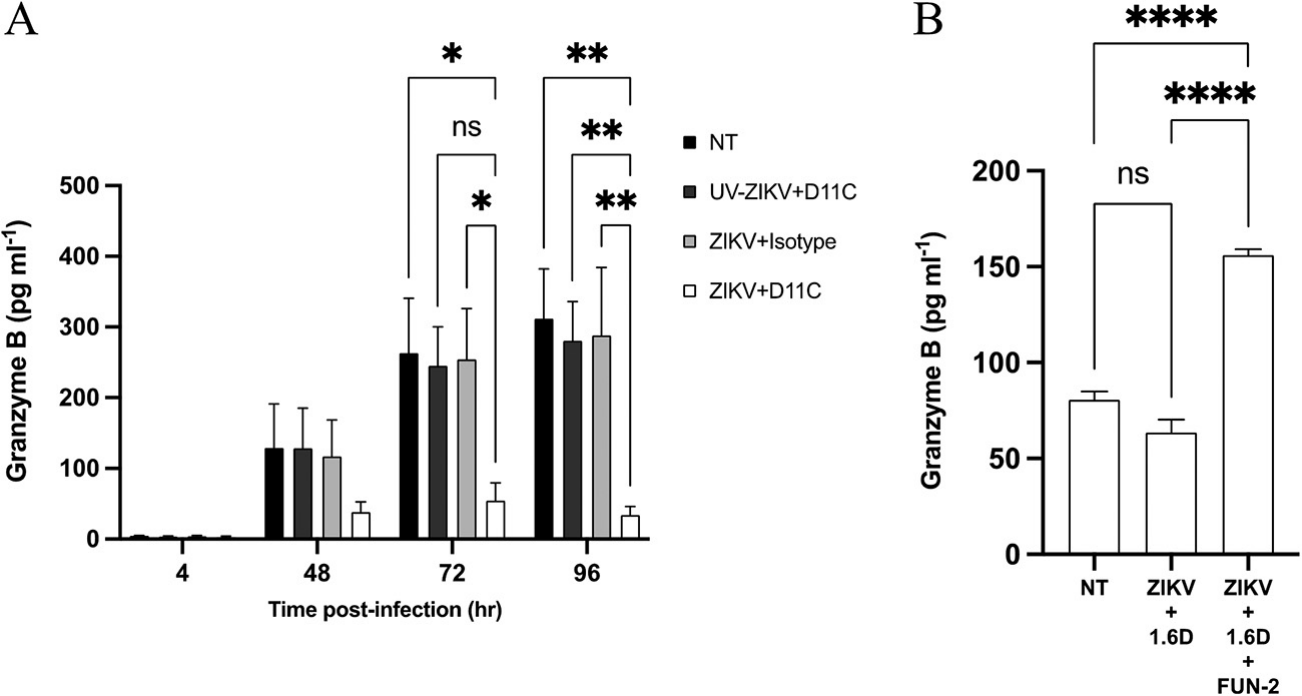
Antibody-dependent enhancement of Zika virus in KU812 cells by DENV antibodies caused reduction of granzyme B secretion. (A) KU812 cells were incubated with ZIKV-PRVABC59 (MOI, 1) for 1 h with either 6 μg/mL D11C or a nonspecific isotype, washed, and resuspended with fresh growth medium. Cell supernatants were collected 4, 48, 72, and 96 hpi. ELISA was performed using the ELLA simplex microfluidics system. A two-way ANOVA and a Tukey’s multiple-comparison test were used to determine whether there was a significant difference in protein release. Data are expressed as PFU/mL ± SEM for *n* = 3 independent experiments, each replicated in triplicate. *, *P* < 0.05; **, *P* < 0.01; ns, nonsignificant. (B) KU812 cells were incubated with either anti-CD32 antibodies (FUN-2) or medium alone and then infected with ZIKV-PRVABC59 (MOI, 1) for 1 h with 12 μg/mL 1.6D, washed, and resuspended with fresh growth medium. Cell supernatants were collected 72 hpi. ELISA was performed using the ELLA simplex microfluidics system. A two-way ANOVA and a Tukey’s multiple-comparison test were used to determine whether there was a significant difference in protein release. Data are expressed as PFU/mL ± SEM for *n* = 3 independent experiments, each replicated in triplicate. *, *P* < 0.05; **, *P* < 0.01; ****, *P* < 0.0001; ns, nonsignificant.

Granzyme B (GrB) is a multifaceted effector molecule notoriously known for its cytotoxic potential. Using a next-generation automated enzyme-linked immunosorbent assay workflow, we show that at 72 hpi, GrB secretion is significantly diminished in KU812 cells infected with ZIKV through D11C relative to that of the isotype control (*P* < 0.05) (Fig. 5A). By 96 hpi, there was a significant decrease in GrB release from KU812 cells infected through antibody-dependent enhancement compared to that of the controls (*P* < 0.01). Additionally, CD32-blocked KU812 cells infected with ZIKV through 1.6D (12 μg/mL) resulted in a significantly higher release of GrB (*P* < 0.0001) (Fig. 5B).

**FIG 6:**
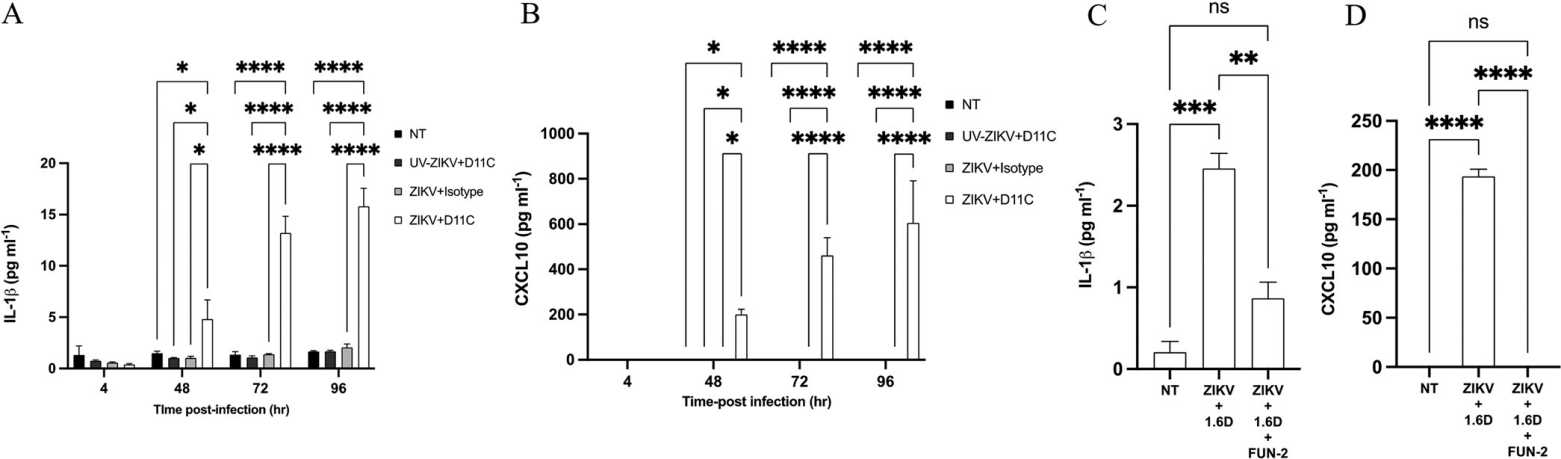
Antibody-dependent enhancement of Zika virus in KU812 cells by DENV antibodies caused release of IL-1β and CXCL10. (A and B) KU812 cells were incubated with ZIKV-PRVABC59 (MOI, 1) for 1 h with either 6 μg/mL D11C or a nonspecific isotype, washed, and resuspended with fresh growth medium. Cell-free supernatants were collected 4, 48, 72, and 96 hpi. ELISA was performed using the ELLA simplex microfluidics system. A two-way ANOVA and a Tukey’s multiple-comparison test were used to determine whether there was a significant difference in protein release. Data are expressed as PFU/mL ± SEM for *n* = 3 independent experiments, each replicated in triplicate. *, *P* < 0.05; **, *P* < 0.01; ***, *P* < 0.001; ****, *P* < 0.0001; ns, nonsignificant. (C and D) KU812 cells were incubated either with anti-CD32 antibodies (FUN-2) or medium alone and then infected with ZIKV-PRVABC59 (MOI, 1) for 1 h with 12 μg/mL 1.6D, washed, and resuspended with fresh growth medium. Cell supernatants were collected 72 hpi. ELISA was performed using the ELLA simplex microfluidics system. A two-way ANOVA and a Tukey’s multiple-comparison test were used to determine whether there was a significant difference in protein release. Data are expressed as PFU/mL ± SEM for *n* = 3 independent experiments, each replicated in triplicate. *, *P* < 0.05; **, *P* < 0.01; ***, *P* < 0.001; ****, *P* < 0.0001; ns, nonsignificant.

IL-1β (27), CXCL10 (33), and CCL5 (26, 32, 33) are established immunological mediators secreted by KU812 cells during DENV infection. Using the same automated enzyme-linked immunosorbent assay workflow, IL-1β and CXCL10 were quantified after ZIKV infection through D11C (6 μg/mL) (Fig. 6A and B). IL-1β and CXCL10 under antibody-dependent enhancement conditions were significantly elevated compared to those of the isotype control at 48 hpi (*P* < 0.05). As time proceeded, there was an accumulation of both mediators 72 hpi and 96 hpi (*P* < 0.0001). There was no difference in protein levels for any conditions 4 hpi (*P* > 0.05). Subsequently, we quantified IL-1β and CXCL10 in CD32-blocked KU812 cell supernatants (Fig. 6C and D). Again, under conditions of viral enhancement, IL-1β and CXCL10 were elevated. However, blocking CD32 resulted in a significant decrease in IL-1β (*P* < 0.01) and a decrease in CXCL10 (*P* < 0.0001).

**FIG 7:**
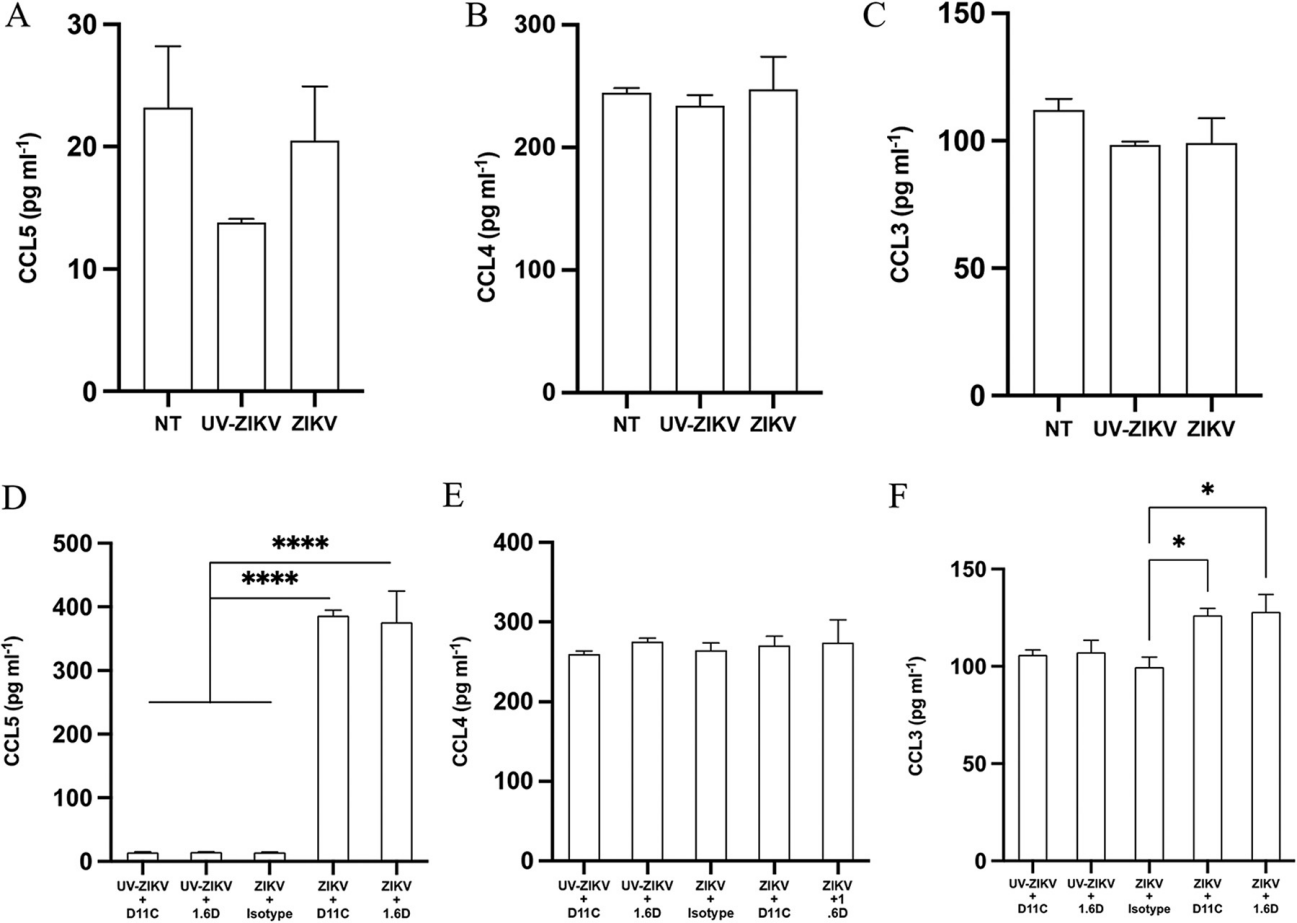
Zika virus infection augmented by cross-reactive dengue virus antibodies in KU812 cells results in significant CCL5 secretion. KU812 cells were infected with infectious or UV-inactivated ZIKV-PRVABC59 (MOI, 1) for 1 h in the presence of a nonspecific isotype control antibody, DENV-specific D11C, or 1.6D (10 μg/mL). Cell-free supernatants were collected 72 hpi, and chemokine secretion was detected by ELISA for (A) CCL5, (B) CCL4, (C) CCL3, (D) CCL5, (E) CCL4, and (F) CCL3. A one-way ANOVA and Tukey’s multiple comparison were used to determine statistical significance. Data are expressed as pg/mL ± SEM for *n* = 3 independent experiments, each replicated in duplicate. *, *P* < 0.05; ****, *P* < 0.0001.

CCL3, CCL4, and CCL5, also known as macrophage inflammatory protein 1α (MIP-1α), MIP-1β, and RANTES, respectively, have previously been identified in the secretome of DENV-infected KU812 cells (26, 33). Enzyme-linked immunosorbent assay (ELISA) was used to quantify chemokine secretion collected from KU812 cells 72 hpi (Fig. 7A to F). Comparing CCL3, 4, and 5 secretion from KU812 cells infected with ZIKV alone compared to that from mock-infected controls, there was no difference between the groups (*P* > 0.05) (Fig. 7A to C). A significant difference in CCL3 secretion (*P* < 0.05) occurred between cells infected with ZIKV-D11C and ZIKV-1.6D immune complexes compared to that of cells infected with ZIKV in the presence of the nonspecific isotype control antibody (Fig. 7F); however, there was not a statistical difference between UV-ZIKV mock-infected KU812 cells and cells infected with immune complexes. A robust secretion of CCL5 was detected in KU812 cells infected with ZIKV-D11C and ZIKV-1.6D immune complexes compared to that of all controls (Fig. 7D). These data establish that CCL5 production is coupled to enhanced ZIKV infection by nonneutralizing DENV antibodies.

## DISCUSSION

KU812 cells have previously been used to establish the first evidence for mast cell/basophil infection by DENV through ADE (27) and subsequently to determine the extent to which vasoactive mediators from DENV-infected mast cells might contribute to dengue hemorrhagic fever and dengue shock syndrome (26). To our knowledge, the characterization of myeloid cells permissive to ZIKV infection has been limited to macrophages (38, 39), monocytes (40, 41), dendritic cells (42), and, most recently, mast cells (37). Additionally, DENV infection extends to myeloid cells to include mast cells and basophils (26, 27). Here, we describe a new model of ZIKV infection—demonstrating the first mast cell/basophil infection by ZIKV—and establish ADE with anti-DENV antibodies coupled to a unique mediator response.

Supernatants of KU812 cells were quantified by plaque assay previously optimized for ZIKV detection (43) to determine the concentration of infectious progeny. Cells infected with ZIKV preincubated with DENV cross-reactive antibodies significantly augmented ZIKV replication. Both anti-DENV—D11C and 1.6D—antibodies had no effect on viral replication when mock infected with UV-inactivated ZIKV (data not shown), confirming that fully functional virus particles are necessary. Importantly, this acted as a dual control, as the presence of D11C and 1.6D antibodies did not contribute to plaque formation or mediator secretion throughout. Compared to that of the isotype control, a 403-fold and 176-fold increase in infection was attributed to D11C and 1.6D, respectively (Fig. 1C). We subsequently determined if viral enhancing effects were consistent across different times (Fig. 2). At 4 hpi, there was no difference in viral replication between ZIKV-D11C and control conditions. Coupled with findings from Fig. 1, KU812 cells are much less permissive to ZIKV infection in an antibody-independent mechanism than in an antibody-dependent mechanism. Low levels of viral replication may prove to be significant in the greater context of ZIKV infection considering ZIKV infection of peripheral blood mononuclear cells can have viral loads ranging from 799 to 16,948 PFU/mL, showing that peripheral blood mononuclear cells can act as a reservoir for ZIKV (44). The greatest antibody-dependent replication effect was detected 48 hpi, after which viral titers began to decline. Previous literature on DENV ADE infection in KU812 cells suggests a peak infection titer between 36 and 72 hpi (26–28). Of note, D11C and 1.6D ZIKV-enhancing capacity in K562 monocytes has previously been established at 72 hpi (15).

Varying MOI had little impact on viral titer under opportune conditions for enhancement (Fig. 3A). This further supports the repeatability of the replication-enhancing potential of this model while also suggesting that the threshold for viral replication under the current conditions can be quite low. Considering that previous work exploring KU812-DENV infection uses MOI ranges from 0.2 to 2 (26, 28), we sought to capture this range and beyond with ZIKV (MOI 0.004 to 4). We also confirm the genetic signature of ZIKV through qPCR analysis performed on the collected cell-free supernatants (Fig. 3B). Genome copy numbers were 100-fold greater than PFU, which is consistent with ZIKV replication in placental tissues in an ADE model (45).

As cross-reactive DENV antibodies form nonneutralizing antibody-virus complexes, the complex is endocytosed after CD32/FcγRII receptor binding. ZIKV replication was significantly reduced when CD32 was blocked prior to adsorption (Fig. 4). These data confirm an antibody-dependent enhancement mechanism of ZIKV replication *in vitro* but should not be inappropriately attributed to severe disease outcomes at the epidemiological level. To our knowledge, though it is hypothesized, there is not yet significant epidemiological data to support enhanced ZIKV disease causality by preexisting DENV virus immunity. However, these data coupled with ZIKV enhancement through monocytes might suggest that ZIKV virions may be aided into cells by circulating DENV antibodies to expand cellular-viral tropism, even if uncoupled to severe disease outcomes.

GrB is a potent cytotoxic mediator released by NK cells and cytotoxic lymphocytes to clear virus-infected cells; however, it has been identified as a mediator in mast cell and basophil biology (46–48). In our ADE model, GrB secretion was significantly reduced, and upon CD32 blocking prior to infection, GrB levels recovered (Fig. 5B). A reduction in GrB production in virus-infected cells might suggest a novel mediator for intrinsic antibody-dependent enhancement, as GrB has not been well characterized in ZIKV-infected innate immune cells via DENV humoral immunity. IL-1β and CXCL10 have previously been identified as immunological mediators released during DENV infection in mast cells (27, 33). Additionally, both mediators have been identified in ZIKV infection (49–53). IL-1β and CXCL10 were significantly elevated in our ADE model here, but when CD32 was blocked, secretion was significantly reduced, tying these mediators to the conditions of ADE. IL-1β has been described as a discriminant score cerebrospinal fluid biomarker in ZIKV-associated microcephalic cases (54). Moreover, DENV-infected mice that lack the CXCL10 receptor have increased mortality rates, as CXCL10 is crucial for CD8^+^ T cell and NK cell recruitment (55) and has also been shown to compete with DENV binding to heparan sulfate (56). However, CXCL10 has been described as the most promising biomarker for acute ZIKV infection with it being involved in fetal neuronal apoptosis and Guillain-Barré syndrome (57).

Similar to mast cells and basophils exclusively infected with DENV, changes in CCL3, CCL4, and CCL5 were not detected in cells infected with ZIKV alone. Contrary to DENV-enhanced KU812-infected cells, we do not report CCL4 secretion in ZIKV-enhanced KU812-infected cells by DENV antibodies. A statistically significant level of CCL3 and CCL5 secretion was detected in antibody-dependent enhanced ZIKV-infected KU812 cells compared to that in nonspecific isotype control-ZIKV-infected cells (Fig. 7). Potent secretion of CCL5 is consistent with previous KU812 chemokine responses to DENV enhancement (26, 27, 32, 33). Considering that adenovirus and respiratory syncytial virus infection in KU812 cells fails to induce CCL5 secretion (26), we postulate that CCL5 might be a selectively induced chemokine in mast cell/basophil-*Flavivirus* infections. Further supporting CCL5 as a mast cell-*Flavivirus* selectively induced chemokine, CCL5 is also significantly upregulated in DENV-infected skin mast cells (58) and human skin fibroblasts (59). Furthermore, DENV nonstructural protein 5 activates CCL5 gene transcription by promoting NF-κB binding to the CCL5 promoter (60, 61). To our knowledge, this mechanism has not been explored in ZIKV, but given the evolutionarily conserved similarities between the viruses, they may well share a similar cellular activation mechanism for CCL5 production. It has been proposed that CCL4, CCL5, and CXCL10 may be beneficial in the context of DENV infection in mast cells (33), and we report that ZIKV induces similar immunological mediator release. Here, we establish that CCL5 production is strongly coupled to enhanced ZIKV infection by nonneutralizing DENV antibodies and offer further support for selective CCL5 secretion as a consequence of immune response to *Flaviviruses.*

**FIG 8:**
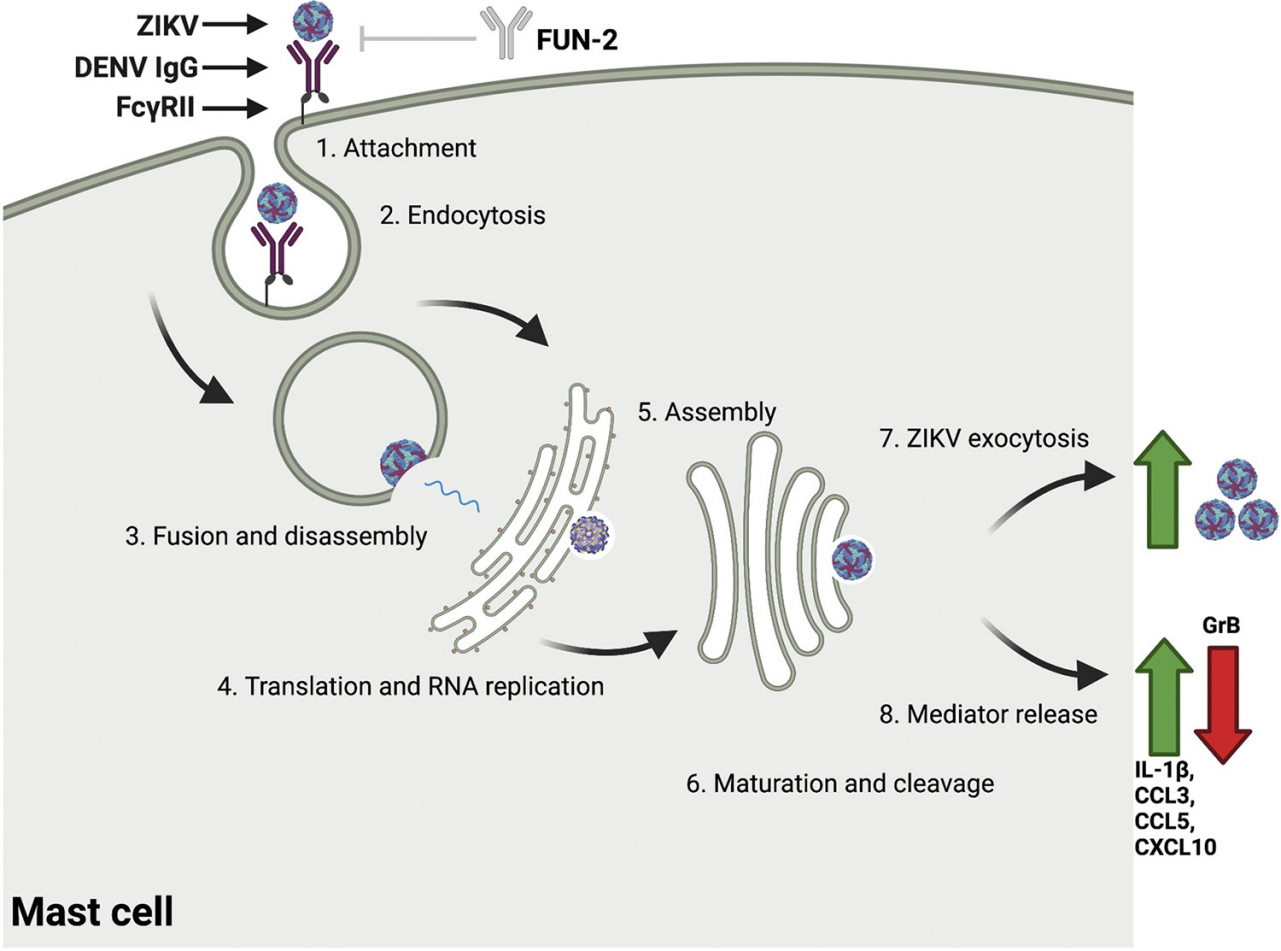
Zika virus replication in a mast cell model is augmented by Dengue virus antibody-dependent enhancement and features a selective immune mediator secretory profile. 1. ZIKV virions attach to the cell surface by cross-reactive DENV IgG antibodies bound to FcγRII (CD32; blocked by FUN-2 anti-FcγRII). 2. ZIKV virions are internalized whereby acidic conditions in the endosome induce conformational changes to the viral E protein enabling viral fusion to the endosomal membrane. 3. Viral nucleocapsid and viral RNA are released into the cytosol. 4. Positive stranded RNA molecule undergoes translation at the endoplasmic reticulum (ER) where a subsequent single polyprotein is then cleaved into structural and nonstructural proteins. 5. Immature virions are assembled in the ER before budding and being transported to the Golgi. 6. Immature virions are transported through the trans-Golgi network where structural changes to the virus take place, including cleavage of prM proteins. 7. The replication cycle is completed as mature virions are liberated from the cell. 8. Selective mediators are secreted from the cell (Image generated with Biorender).

KU812 mast cells/basophils have been integral in determining mast cell permissiveness to DENV infection along with mast cell immune-related responses. We further the understanding of mast cell/basophil-*Flavivirus* interactions and show that ZIKV infects KU812 cells independent of a cross-reactive antibody, albeit at relatively low levels; however, ZIKV infection is significantly enhanced in the presence of a cross-reactive DENV antibody. Additionally, ADE of ZIKV is coupled to selective CCL5, IL-1β, and CXCL10 secretion (Fig. 8), further supporting the role of *Flavivirus-*inducible mediators. As others have suggested in mast cell-dengue virus identification studies, the results of this study support further investigation to identify the full spectrum of cell types which are infected by ZIKV and their contributions to shaping the resultant immune milieu.

## MATERIALS AND METHODS

### Antibodies.

Previously characterized immunoglobulin G1 kappa (IgG1-κ) anti-DENV human monoclonal antibodies (hMAbs), D11C and 1.6D (15, 62), were kindly provided by JS Schieffelin (Tulane University, USA). Human purified IgG1-κ isotype control was purchased from Millipore Sigma (Millipore Sigma, I5154).

### Cell culture.

Briefly, Vero E6 cells (ATCC CRL1586) were grown in Dulbecco modified Eagle medium (DMEM) supplemented with 10% fetal bovine serum (FBS) and 1% penicillin streptomycin. Human chronic myelogenous leukemia KU812 cells were cultured as described previously (28).

### Zika virus propagation and UV-inactivation.

Vero E6 cells were infected (MOI, 0.1) with PRVABC59-ZIKV (ATCC VR1843) at 85% confluence and incubated for 1 h at 37°C and 5% CO_2_. Viral supernatant was removed, and cells were washed with phosphate-buffered saline (PBS). Complete medium was added atop the monolayer, and ZIKV was propagated for 5 days. Viral supernatant was harvested, and titer was determined by plaque assay. After viral propagation, viral stock aliquots were thawed and added to one well of a 6-well tissue culture plate. The base of the plate was covered in foil and the lid removed. The dish was then suspended on a platform within 30 cm of a standard UVC light within a biosafety cabinet. The virus was exposed to UVC light for 1 h, and inactivation was confirmed by the absence of plaques in a plaque formation assay.

### ZIKV infection assay and CD32 blocking.

Briefly, PRVABC59-ZIKV was incubated at 37°C and 5% CO_2_ for 1 h with hMAbs (6 or 10 μg/mL final concentration) and medium. KU812 cells (160,000 cells/mL) at a final volume of 2 mL were combined with the antibody-ZIKV mixtures and incubated (MOI, 4.0 × 10^−3^ to 4) for 1 h at 37°C and 5% CO_2_. Cells were then centrifuged (300 × *g*), viral supernatant was discarded, and pellet was washed and then resuspended with 2 mL of fresh medium. Supernatant was collected 4, 48, 72, and 96 h postinfection (hpi). CD32 blocking assay was performed identically, except prior to infection through 1.6D (12 μg/mL), cells were incubated with an FcγRII block (FUN-2; 50 μg/mL; Biolegend, San Diego, CA).

### Plaque assay.

Vero E6 cells were infected at 85% confluence in 6-well tissue culture plates (1:10 to 1:100,000 dilution series) of supernatant harvested from KU812 cells 72 hpi. Vero E6 cells were incubated at 37°C and 5% CO_2_ for 1 h before supernatant was removed and cells were washed with PBS. Subsequently, 2 mL of equal carboxymethyl cellulose (CMC)/DMEM mixture overlay (supplemented with 5% FBS and 1% penicillin/streptomycin) was added atop the monolayer and incubated for 5 days at 37°C and 5% CO_2_. Overlay was removed, Vero E6 cells were washed with PBS, and cells were stained with crystal violet (Millipore Sigma, C0775). Infectious virus was quantified by the average number of plaques formed per milliliter of sample.

### ELISA.

CCL3, CCL4, and CCL5 in cell-free supernatants were quantified using DuoSet ELISA kits purchased from R&D systems (Minneapolis, MN) according to the manufacturer’s instructions. IL-1β, CXCL10, and GrB in cell-free supernatants were quantified using simple plex assay kits purchased from Protein Simple (Minneapolis, MN) and analyzed on on an ELLA next generation microfluidics platform (Protein Simple) according to the manufacturer’s instructions and using Simple Plex software Runner and Explorer.

### Real-time PCR.

Cell-free viral supernatants were collected, and viral RNA was extracted using Qiagen RNeasy plus kit (Qiagen, Hilden, Germany) according to the manufacturer’s instructions. Isolated RNA was reverse transcribed to cDNA with EcoDry RNA to double-primed reverse transcriptase (Clontech, Mountain View, CA). Optimal primer amplification efficiency for ZIKV For 5′-GCAAACGCGGTCGCAAACCT-3′ and Rev 5′-TGCTAACGCGAAGCCAGGGT-3′ were carried out prior to quantification (IDT, Coralville, IA). ABI StepOnePlus real-time PCR instrument was used to perform quantitative PCR. A standard curve was generated using gBlock gene fragments (IDT, Coralville, IA) to determine number of ZIKV copies in cell-free supernatant.

### Cell viability assay.

KU812 cells (2 × 10^5/^well) in a final volume of 100 μL were seeded in a 96-well tissue culture plate. Cells were incubated with 50 μg/mL FUN-2 or medium control at 37°C and 5% CO_2_ for 1 h. After incubation, 10 μL/well WST-1 cell proliferation reagent (Sigma-Aldrich, St. Louis, MO) was added. Following a 4-h incubation, samples were analyzed with a spectrophotometer (Synergy, BIO-TEK) at a 470-nm wavelength.

### Statistical analysis.

All data analysis and graphs were prepared with GraphPad Prism 8 (GraphPad Software, San Diego, CA) for Mac OS. When comparing multiple groups, a one-way analysis of variance (ANOVA) and Tukey or Dunnett’s multiple comparisons were used. When comparing 2 groups, a two-tailed paired Student’s *t* test was performed to determine if there was a statistical difference. In all cases, a probability value less than 0.05 was interpreted as statistically significant.

## References

[1] Hawkes RA. 1964. Enhancement of the infectivity of arboviruses by specific antisera produced in domestic fowls. Aust J Exp Biol Med Sci 42:465–482. doi:10.1038/icb.1964.44. 14202187

[2] Halstead SB, Chow JS, Marchette NJ. 1973. Immunological enhancement of dengue virus replication. Nat New Biol 243:24–26. 17319077

[3] Katzelnick LC, Gresh L, Halloran ME, Mercado JC, Kuan G, Gordon A, Balmaseda A, Harris E. 2017. Antibody-dependent enhancement of severe dengue disease in humans. Science 358:929–932. doi:10.1126/science.aan6836. 29097492 PMC5858873

[4] Langerak T, Mumtaz N, Tolk VI, van Gorp ECM, Martina BE, Rockx B, Koopmans MPG. 2019. The possible role of cross-reactive dengue virus antibodies in Zika virus pathogenesis. PLoS Pathog 15:e1007640. doi:10.1371/journal.ppat.1007640. 30998804 PMC6472811

[5] Baloch Z, Shen Z, Zhang L, Feng Y, Li D, Zhang N-N, Deng Y-Q, Yang C, Sun X, Dai J, Yang Z, Qin C-F, Xia X. 2021. Recapitulating Zika virus infection in vagina of tree shrew (Tupaia belangeri). Front Cell Infect Microbiol 11:687338. doi:10.3389/fcimb.2021.687338. 34249779 PMC8270636

[6] Wen J, Shresta S. 2019. Antigenic cross-reactivity between Zika and dengue viruses: is it time to develop a universal vaccine? Curr Opin Immunol 59:1–8. doi:10.1016/j.coi.2019.02.001. 30884384 PMC6745280

[7] Priyamvada L, Quicke KM, Hudson WH, Onlamoon N, Sewatanon J, Edupuganti S, Pattanapanyasat K, Chokephaibulkit K, Mulligan MJ, Wilson PC, Ahmed R, Suthar MS, Wrammert J. 2016. Human antibody responses after dengue virus infection are highly cross-reactive to Zika virus. Proc Natl Acad Sci USA 113:7852–7857. doi:10.1073/pnas.1607931113. 27354515 PMC4948328

[8] Dejnirattisai W, Supasa P, Wongwiwat W, Rouvinski A, Barba-Spaeth G, Duangchinda T, Sakuntabhai A, Cao-Lormeau V-M, Malasit P, Rey FA, Mongkolsapaya J, Screaton GR. 2016. Dengue virus sero-cross-reactivity drives antibody-dependent enhancement of infection with Zika virus. Nat Immunol 17:1102–1108. doi:10.1038/ni.3515. 27339099 PMC4994874

[9] Swanstrom JA, Plante JA, Plante KS, Young EF, McGowan E, Gallichotte EN, Widman DG, Heise MT, de Silva AM, Baric RS. 2016. Dengue virus envelope dimer epitope monoclonal antibodies isolated from dengue patients are protective against Zika virus. mBio 7. doi:10.1128/mBio.01123-16. PMC4958264 27435464

[10] Huber RG, Lim XN, Ng WC, Sim AYL, Poh HX, Shen Y, Lim SY, Sundstrom KB, Sun X, Aw JG, Too HK, Boey PH, Wilm A, Chawla T, Choy MM, Jiang L, de Sessions PF, Loh XJ, Alonso S, Hibberd M, Nagarajan N, Ooi EE, Bond PJ, Sessions OM, Wan Y. 2019. Structure mapping of dengue and Zika viruses reveals functional long-range interactions. Nat Commun 10:1408. doi:10.1038/s41467-019-09391-8. 30926818 PMC6441010

[11] Gunawardana SA, Shaw RH. 2018. Cross-reactive dengue virus-derived monoclonal antibodies to Zika virus envelope protein: Panacea or Pandora’s box? BMC Infect Dis 18:641. doi:10.1186/s12879-018-3572-0. 30526531 PMC6288897

[12] Bardina SV, Bunduc P, Tripathi S, Duehr J, Frere JJ, Brown JA, Nachbagauer R, Foster GA, Krysztof D, Tortorella D, Stramer SL, García-Sastre A, Krammer F, Lim JK. 2017. Enhancement of Zika virus pathogenesis by preexisting antiflavivirus immunity. Science 356:175–180. doi:10.1126/science.aal4365. 28360135 PMC5714274

[13] Slon-Campos JL, Dejnirattisai W, Jagger BW, López-Camacho C, Wongwiwat W, Durnell LA, Winkler ES, Chen RE, Reyes-Sandoval A, Rey FA, Diamond MS, Mongkolsapaya J, Screaton GR. 2019. A protective Zika virus E-dimer-based subunit vaccine engineered to abrogate antibody-dependent enhancement of dengue infection. Nat Immunol 20:1291–1298. doi:10.1038/s41590-019-0477-z. 31477918 PMC6839414

[14] Camargos VN, Foureaux G, Medeiros DC, da Silveira VT, Queiroz-Junior CM, Matosinhos ALB, Figueiredo AFA, Sousa CDF, Moreira TP, Queiroz VF, Dias ACF, Santana KTO, Passos I, Real ALCV, Silva LC, Mourão FAG, Wnuk NT, Oliveira MAP, Macari S, Silva T, Garlet GP, Jackman JA, Soriani FM, Moraes MFD, Mendes EMAM, Ribeiro FM, Costa GMJ, Teixeira AL, Cho N-J, Oliveira ACP, Teixeira MM, Costa VV, Souza DG. 2019. In-depth characterization of congenital Zika syndrome in immunocompetent mice: antibody-dependent enhancement and an antiviral peptide therapy. EBioMedicine 44:516–529. doi:10.1016/j.ebiom.2019.05.014. 31130472 PMC6604363

[15] Paul LM, Carlin ER, Jenkins MM, Tan AL, Barcellona CM, Nicholson CO, Michael SF, Isern S. 2016. Dengue virus antibodies enhance Zika virus infection. Clin Transl Immunology 5:e117. doi:10.1038/cti.2016.72. PMC5192063 28090318

[16] Rathore APS, Saron WAA, Lim T, Jahan N, St John AL. 2019. Maternal immunity and antibodies to dengue virus promote infection and Zika virus-induced microcephaly in fetuses. Sci Adv 5:eaav3208. doi:10.1126/sciadv.aav3208. 30820456 PMC6392794

[17] Brown JA, Singh G, Acklin JA, Lee S, Duehr JE, Chokola AN, Frere JJ, Hoffman KW, Foster GA, Krysztof D, Cadagan R, Jacobs AR, Stramer SL, Krammer F, García-Sastre A, Lim JK. 2019. Dengue virus immunity increases Zika virus-induced damage during pregnancy. Immunity 50:751–762.e5. doi:10.1016/j.immuni.2019.01.005. 30737148 PMC6947917

[18] Pedroso C, Fischer C, Feldmann M, Sarno M, Luz E, Moreira-Soto A, Cabral R, Netto EM, Brites C, Kümmerer BM, Drexler JF. 2019. Cross-protection of dengue virus infection against congenital Zika syndrome, Northeastern Brazil. Emerg Infect Dis 25:1485–1493. doi:10.3201/eid2508.190113. 31075077 PMC6649334

[19] Kam Y-W, Lee CY-P, Teo T-H, Howland SW, Amrun SN, Lum F-M, See P, Kng NQ-R, Huber RG, Xu M-H, Tan H-L, Choo A, Maurer-Stroh S, Ginhoux F, Fink K, Wang C-I, Ng LFP, Rénia L. 2017. Cross-reactive dengue human monoclonal antibody prevents severe pathologies and death from Zika virus infections. JCI Insight 2. doi:10.1172/jci.insight.92428. PMC5396524 28422757

[20] Collins MH, McGowan E, Jadi R, Young E, Lopez CA, Baric RS, Lazear HM, de Silva AM. 2017. Lack of durable cross-neutralizing antibodies against Zika virus from dengue virus infection. Emerg Infect Dis 23:773–781. doi:10.3201/eid2305.161630. 28418292 PMC5403059

[21] Hepworth MR, Maurer M, Hartmann S. 2012. Regulation of type 2 immunity to helminths by mast cells. Gut Microbes 3:476–481. doi:10.4161/gmic.21507. 22892692 PMC3467025

[22] St John AL, Rathore APS, Yap H, Ng M-L, Metcalfe DD, Vasudevan SG, Abraham SN. 2011. Immune surveillance by mast cells during dengue infection promotes natural killer (NK) and NKT-cell recruitment and viral clearance. Proc Natl Acad Sci USA 108:9190–9195. doi:10.1073/pnas.1105079108. 21576486 PMC3107258

[23] Demeure CE, Brahimi K, Hacini F, Marchand F, Péronet R, Huerre M, St-Mezard P, Nicolas J-F, Brey P, Delespesse G, Mécheri S. 2005. Anopheles mosquito bites activate cutaneous mast cells leading to a local inflammatory response and lymph node hyperplasia. J Immunol 174:3932–3940. doi:10.4049/jimmunol.174.7.3932. 15778349

[24] Blom T, Huang R, Aveskogh M, Nilsson K, Hellman L. 1992. Phenotypic characterization of KU812, a cell line identified as an immature human basophilic leukocyte. Eur J Immunol 22:2025–2032. doi:10.1002/eji.1830220811. 1639103

[25] Kishi K. 1985. A new leukemia cell line with Philadelphia chromosome characterized as basophil precursors. Leuk Res 9:381–390. doi:10.1016/0145-2126(85)90060-8. 3858609

[26] King CA, Anderson R, Marshall JS. 2002. Dengue virus selectively induces human mast cell chemokine production. J Virol 76:8408–8419. doi:10.1128/JVI.76.16.8408-8419.2002. 12134044 PMC155122

[27] King CA, Marshall JS, Alshurafa H, Anderson R. 2000. Release of vasoactive cytokines by antibody-enhanced dengue virus infection of a human mast cell/basophil line. J Virol 74:7146–7150. doi:10.1128/jvi.74.15.7146-7150.2000. 10888655 PMC112233

[28] Brown MG, Huang YY, Marshall JS, King CA, Hoskin DW, Anderson R. 2009. Dramatic caspase-dependent apoptosis in antibody-enhanced dengue virus infection of human mast cells. J Leukoc Biol 85:71–80. doi:10.1189/jlb.0308167. 18809735

[29] Fang Y-T, Wan S-W, Lu Y-T, Yao J-H, Lin C-F, Hsu L-J, Brown MG, Marshall JS, Anderson R, Lin Y-S. 2014. Autophagy facilitates antibody-enhanced dengue virus infection in human pre-basophil/mast cells. PLoS One 9:e110655. doi:10.1371/journal.pone.0110655. 25329914 PMC4199741

[30] Furuta T, Murao LA, Lan NTP, Huy NT, Huong VTQ, Thuy TT, Tham VD, Nga CTP, Ha TTN, Ohmoto Y, Kikuchi M, Morita K, Yasunami M, Hirayama K, Watanabe N. 2012. Association of mast cell-derived VEGF and proteases in Dengue shock syndrome. PLoS Negl Trop Dis 6:e1505. doi:10.1371/journal.pntd.0001505. 22363824 PMC3283553

[31] Fu Y, Chen Y-L, Herve M, Gu F, Shi P-Y, Blasco F. 2014. Development of a FACS-based assay for evaluating antiviral potency of compound in dengue infected peripheral blood mononuclear cells. J Virol Methods 196:18–24. doi:10.1016/j.jviromet.2013.09.009. 24140514

[32] Brown MG, King CA, Sherren C, Marshall JS, Anderson R. 2006. A dominant role for FcgammaRII in antibody-enhanced dengue virus infection of human mast cells and associated CCL5 release. J Leukoc Biol 80:1242–1250. doi:10.1189/jlb.0805441. 16940332

[33] Brown MG, McAlpine SM, Huang YY, Haidl ID, Al-Afif A, Marshall JS, Anderson R. 2012. RNA sensors enable human mast cell anti-viral chemokine production and IFN-mediated protection in response to antibody-enhanced dengue virus infection. PLoS One 7:e34055. doi:10.1371/journal.pone.0034055. 22479521 PMC3316603

[34] Khandia R, Munjal A, Dhama K, Karthik K, Tiwari R, Malik YS, Singh RK, Chaicumpa W. 2018. Modulation of dengue/Zika virus pathogenicity by antibody-dependent enhancement and strategies to protect against enhancement in Zika virus infection. Front Immunol 9:597. doi:10.3389/fimmu.2018.00597. 29740424 PMC5925603

[35] Londono-Renteria B, Marinez-Angarita JC, Troupin A, Colpitts TM. 2017. Role of mast cells in dengue virus pathogenesis. DNA Cell Biol 36:423–427. doi:10.1089/dna.2017.3765. 28486041

[36] Coish JM, Crozier RWE, Schieffelin JS, Coorssen JR, Hunter FF, MacNeil AJ. 2020. Mast cell infection by Zika virus and augmentation by pre-existing dengue virus immunity. FASEB J 34. doi:10.1096/fasebj.2020.34.s1.04535.

[37] Rabelo K, da Silva Gonçalves AJ, de Souza LJ, Sales AP, de Lima SMB, Trindade GF, Ciambarella BT, Amorim Tasmo NR, Diaz BL, de Carvalho JJ, de Oliveira Duarte MP, Paes MV. 2020. Zika virus infects human placental mast cells and the HMC-1 cell line, and triggers degranulation, cytokine release and ultrastructural changes. Cells 9:975. doi:10.3390/cells9040975. 32316163 PMC7227014

[38] Quicke KM, Bowen JR, Johnson EL, McDonald CE, Ma H, O'Neal JT, Rajakumar A, Wrammert J, Rimawi BH, Pulendran B, Schinazi RF, Chakraborty R, Suthar MS. 2016. Zika virus infects human placental macrophages. Cell Host Microbe 20:83–90. doi:10.1016/j.chom.2016.05.015. 27247001 PMC5166429

[39] Jurado KA, Simoni MK, Tang Z, Uraki R, Hwang J, Householder S, Wu M, Lindenbach BD, Abrahams VM, Guller S, Fikrig E. 2016. Zika virus productively infects primary human placenta-specific macrophages. JCI Insight 1. doi:10.1172/jci.insight.88461. PMC5007065 27595140

[40] Khaiboullina SF, Uppal T, Sarkar R, Gorzalski A, St Jeor S, Verma SC. 2017. ZIKV infection regulates inflammasomes pathway for replication in monocytes. Sci Rep 7:16050. doi:10.1038/s41598-017-16072-3. 29167459 PMC5700238

[41] Yoshikawa FSY, Pietrobon AJ, Branco ACCC, Pereira NZ, da Silva Oliveira LM, Machado CM, da Silva Duarte AJ, Sato MN. 2019. Zika virus infects newborn monocytes without triggering a substantial cytokine response. J Infect Dis 220:32–40. doi:10.1093/infdis/jiz075. 30785182

[42] Vielle NJ, Zumkehr B, García-Nicolás O, Blank F, Stojanov M, Musso D, Baud D, Summerfield A, Alves MP. 2018. Silent infection of human dendritic cells by African and Asian strains of Zika virus. Sci Rep 8:5440. doi:10.1038/s41598-018-23734-3. 29615676 PMC5882923

[43] Agbulos DS, Barelli L, Giordano BV, Hunter FF. 2016. Virus: quantification, propagation, detection, and storage. Curr Protoc Microbiol 43:15D.4.1–15D.4.16. 10.1002/cpmc.19 27858969

[44] Messias CV, Lemos JP, Cunha DP, Vasconcelos Z, Raphael LMS, Bonaldo MC, Cister-Alves B, Bou-Habib DC, Cotta-de-Almeida V, Savino W, Mendes-da-Cruz DA. 2019. Zika virus infects human blood mononuclear cells. BMC Infect Dis 19:986. doi:10.1186/s12879-019-4622-y. 31752731 PMC6873492

[45] Hermanns K, Göhner C, Kopp A, Schmidt A, Merz WM, Markert UR, Junglen S, Drosten C. 2018. Zika virus infection in human placental tissue explants is enhanced in the presence of dengue virus antibodies in-vitro. Emerg Microbes Infect 7:198. doi:10.1038/s41426-018-0199-6. 30504926 PMC6274641

[46] Pardo J, Wallich R, Ebnet K, Iden S, Zentgraf H, Martin P, Ekiciler A, Prins A, Müllbacher A, Huber M, Simon MM. 2007. Granzyme B is expressed in mouse mast cells in vivo and in vitro and causes delayed cell death independent of perforin. Cell Death Differ 14:1768–1779. doi:10.1038/sj.cdd.4402183. 17599099

[47] Strik MCM, de Koning PJA, Kleijmeer MJ, Bladergroen BA, Wolbink AM, Griffith JM, Wouters D, Fukuoka Y, Schwartz LB, Hack CE, van Ham SM, Kummer JA. 2007. Human mast cells produce and release the cytotoxic lymphocyte associated protease granzyme B upon activation. Mol Immunol 44:3462–3472. doi:10.1016/j.molimm.2007.03.024. 17485116

[48] Tschopp CM, Spiegl N, Didichenko S, Lutmann W, Julius P, Virchow JC, Hack CE, Dahinden CA. 2006. Granzyme B, a novel mediator of allergic inflammation: its induction and release in blood basophils and human asthma. Blood 108:2290–2299. doi:10.1182/blood-2006-03-010348. 16794249

[49] Zuñiga J, Choreño-Parra JA, Jiménez-Alvarez L, Cruz-Lagunas A, Márquez-García JE, Ramírez-Martínez G, Goodina A, Hernández-Montiel E, Fernández-López LA, Cabrera-Cornejo MF, Cabello C, Castillejos M, Hernández A, Regino-Zamarripa NE, Mendoza-Milla C, Vivanco-Cid H, Escobar-Gutierrez A, Fonseca-Coronado S, Belaunzarán-Zamudio PF, Pérez-Patrigeon S, Guerrero L, Regalado J, Nájera-Cancino G, Caballero-Sosa S, Rincón-León H, Smolskis M, Mateja A, Hunsberger S, Beigel JH, Ruiz-Palacios G, Emerging Infectious Diseases Clinical Research Network of Mexico (La Red). 2020. A unique immune signature of serum cytokine and chemokine dynamics in patients with Zika virus infection from a tropical region in Southern Mexico. Int J Infect Dis 94:4–11. doi:10.1016/j.ijid.2020.02.014. 32081772 PMC7362833

[50] Tappe D, Pérez-Girón JV, Zammarchi L, Rissland J, Ferreira DF, Jaenisch T, Gómez-Medina S, Günther S, Bartoloni A, Muñoz-Fontela C, Schmidt-Chanasit J. 2016. Cytokine kinetics of Zika virus-infected patients from acute to reconvalescent phase. Med Microbiol Immunol 205:269–273. doi:10.1007/s00430-015-0445-7. 26702627 PMC4867002

[51] Wang J, Liu J, Zhou R, Ding X, Zhang Q, Zhang C, Li L. 2018. Zika virus infected primary microglia impairs NPCs proliferation and differentiation. Biochem Biophys Res Commun 497:619–625. doi:10.1016/j.bbrc.2018.02.118. 29453985

[52] Wang W, Li G, Wu D, Luo Z, Pan P, Tian M, Wang Y, Xiao F, Li A, Wu K, Liu X, Rao L, Liu F, Liu Y, Wu J. 2018. Zika virus infection induces host inflammatory responses by facilitating NLRP3 inflammasome assembly and interleukin-1β secretion. Nat Commun 9:106. doi:10.1038/s41467-017-02645-3. 29317641 PMC5760693

[53] Lum F-M, Lye DCB, Tan JJL, Lee B, Chia P-Y, Chua T-K, Amrun SN, Kam Y-W, Yee W-X, Ling W-P, Lim VWX, Pang VJX, Lee LK, Mok EWH, Chong C-Y, Leo Y-S, Ng LFP. 2018. Longitudinal study of cellular and systemic cytokine signatures to define the dynamics of a balanced immune environment during disease manifestation in Zika virus–infected patients. J Infect Dis 218:814–824. doi:10.1093/infdis/jiy225. 29672707 PMC6057545

[54] Nascimento-Carvalho GC, Nascimento-Carvalho EC, Ramos CL, Vilas-Boas A-L, Moreno-Carvalho OA, Vinhaes CL, Barreto-Duarte B, Queiroz ATL, Andrade BB, Nascimento-Carvalho CM. 2021. Zika-exposed microcephalic neonates exhibit higher degree of inflammatory imbalance in cerebrospinal fluid. Sci Rep 11:8474. doi:10.1038/s41598-021-87895-4. 33875756 PMC8055905

[55] Hsieh M-F, Lai S-L, Chen J-P, Sung J-M, Lin Y-L, Wu-Hsieh BA, Gerard C, Luster A, Liao F. 2006. Both CXCR3 and CXCL10/IFN-inducible protein 10 are required for resistance to primary infection by dengue virus. J Immunol 177:1855–1863. doi:10.4049/jimmunol.177.3.1855. 16849497

[56] Chen J-P, Lu H-L, Lai S-L, Campanella GS, Sung J-M, Lu M-Y, Wu-Hsieh BA, Lin Y-L, Lane TE, Luster AD, Liao F. 2006. Dengue virus induces expression of CXC chemokine ligand 10/IFN-γ-inducible protein 10, which competitively inhibits viral binding to cell surface heparan sulfate. J Immunol 177:3185–3192. doi:10.4049/jimmunol.177.5.3185. 16920957

[57] Naveca FG, Pontes GS, Chang AY-H, da Silva GAV, do Nascimento VA, da Silva Monteiro DC, da Silva MS, Abdalla LF, Santos JHA, de Almeida TAP, Mejía MDCC, de Mesquita TGR, de Souza Encarnação HV, de Souza Gomes M, Amaral LR, Campi-Azevedo AC, Coelho-Dos-Reis JG, do Vale Antonelli LR, Teixeira-Carvalho A, Martins-Filho OA, Ramasawmy R. 2018. Analysis of the immunological biomarker profile during acute Zika virus infection reveals the overexpression of CXCL10, a chemokine linked to neuronal damage. Mem Inst Oswaldo Cruz 113:e170542. 29768624 10.1590/0074-02760170542 PMC5961926

[58] Troupin A, Shirley D, Londono-Renteria B, Watson AM, McHale C, Hall A, Hartstone-Rose A, Klimstra WB, Gomez G, Colpitts TM. 2016. A role for human skin mast cells in dengue virus infection and systemic spread. J Immunol 197:4382–4391. doi:10.4049/jimmunol.1600846. 27799312

[59] Hamel R, Dejarnac O, Wichit S, Ekchariyawat P, Neyret A, Luplertlop N, Perera-Lecoin M, Surasombatpattana P, Talignani L, Thomas F, Cao-Lormeau V-M, Choumet V, Briant L, Desprès P, Amara A, Yssel H, Missé D. 2015. Biology of Zika virus infection in human skin cells. J Virol 89:8880–8896. doi:10.1128/JVI.00354-15. 26085147 PMC4524089

[60] Khunchai S, Junking M, Suttitheptumrong A, Kooptiwut S, Haegeman G, Limjindaporn T, Yenchitsomanus P-T. 2015. NF-κB is required for dengue virus NS5-induced RANTES expression. Virus Res 197:92–100. doi:10.1016/j.virusres.2014.12.007. 25523420

[61] Khunchai S, Junking M, Suttitheptumrong A, Yasamut U, Sawasdee N, Netsawang J, Morchang A, Chaowalit P, Noisakran S, Yenchitsomanus P-T, Limjindaporn T. 2012. Interaction of dengue virus nonstructural protein 5 with Daxx modulates RANTES production. Biochem Biophys Res Commun 423:398–403. doi:10.1016/j.bbrc.2012.05.137. 22664104

[62] Costin JM, Zaitseva E, Kahle KM, Nicholson CO, Rowe DK, Graham AS, Bazzone LE, Hogancamp G, Figueroa Sierra M, Fong RH, Yang S-T, Lin L, Robinson JE, Doranz BJ, Chernomordik LV, Michael SF, Schieffelin JS, Isern S. 2013. Mechanistic study of broadly neutralizing human monoclonal antibodies against dengue virus that target the fusion loop. J Virol 87:52–66. doi:10.1128/JVI.02273-12. 23077306 PMC3536401

